# Incidence and predictors of Tuberculosis among patients enrolled in Anti-Retroviral Therapy after universal test and treat program, Addis Ababa, Ethiopia. A retrospective follow -up study

**DOI:** 10.1371/journal.pone.0272358

**Published:** 2022-08-03

**Authors:** Amare Getu, Haileab Fekadu Wolde, Yaregal Animut, Anteneh Ayelign Kibret

**Affiliations:** 1 Ethiopian Public Health Institute, Addis Ababa, Ethiopia; 2 Department of Epidemiology and Biostatistics, Institute of Public Health, College of Medicine and Health Sciences, University of Gondar, Gondar, Ethiopia; 3 Department of Human Anatomy, School of Medicine, College of Medicine and Health Sciences, University of Gondar, Gondar, Ethiopia; The University of Georgia, UNITED STATES

## Abstract

**Introduction:**

Tuberculosis (TB) is the leading killer of people living with HIV (PLHIV) and almost one-third of deaths in the world are attributed to it and many of these deaths occur in developing countries. Despite these evidences, after the implementation of universal test and treat (UTT) strategy, information regarding the incidence and predictors of tuberculosis among PLHIV is limited in Ethiopia. Therefore, this study aimed to assess the incidence and predictors of tuberculosis among patients enrolled in Anti-Retroviral Therapy (ART) after universal test and treat program at St. Peter hospital and Zewditu Memorial Hospital, Addis Ababa, Ethiopia.

**Methods:**

Institutional-based retrospective cohort study was conducted from November 1 to 30, 2020. Simple random sampling was used to select a total of 539 adults records which was enrolled on ART. Data was collected and entered into EPI DATA 3.1 and analyzed using STATA version 14.1. Time-to-event distributions were estimated using Kaplan–Meier estimates. Hazards across different categories were compared using log-rank tests. Predictors were identified using the Cox proportional hazards model. The hazard ratio (HR) and 95% confidence interval (CI) were computed. Variables having P-value < 0.05 from the multivariable analysis were considered as a statistically significant.

**Result:**

Among 539 records reviewed, 529 (98%) were included in the final analysis. The total follow-up period was 1529 Person-Year (PY). The incidence rate in this cohort was found to be 4.84 per 100-person year (95%CI,3.83–6.11). CD4 count<200 (AHR: 3.14,95% CI:1.64–7.10), poor adherence (AHR:2.16, 95% CI:1.21–3.85), underweight (AHR:2.42, 95% CI: 1.30–4.51), not taking isoniazid prophylaxis therapy (AHR: 2.78,95% CI: 1.06–7.30), being bedridden 3.06; (AHR: 3.06, 95% CI: 1.50–6.24), and baseline WHO stage three or four (AHR:2.33, 95% CI:1.08–5.02) were independent predictors for the incidence of TB among HIV positive patients.

**Conclusion:**

In this study, the incidence of tuberculosis is relatively low as compared to studies done before the initiation of test and treat program in Ethiopia. low CD4count, poor level of adherence, low BMI, not taking IPT prophylaxis, bedridden functional status, and being on baseline WHO stage III or IV were found to increase the hazard of tuberculosis. Hence, close follow up, reminders, surveillance, and tracing mechanisms targeting this higher risk group would decrease Tuberculosis among PLHIV.

## Introduction

Tuberculosis (TB) is one of the public health burdens and the leading cause of morbidity and mortality among HIV positive patients. *Mycobacterium tuberculosis* (MTB) is a causative agent for TB and mainly affects the lungs but also involve almost all body organs [1,2]. World Health Organization (WHO) depict that around 4 billion deaths per year is recorded due to TB and HIV Co-infection [3]. Among all TB cases, 8.6% were people living with HIV and the rate of progression is much higher at about 10–15% per year [1,3,4]. Approximately 1.1 million peoples are living with TB and HIV/AIDS worldwide and from them 80% are living in Sub-Saharan countries [3]. A recent report by UNAIDS revealed that more than 75% of all estimated HIV-TB co-infected individuals live in just 10 countries, and nine of those countries are in SSA [5]. Even though, TB-related deaths among people living with HIV (PLHIV) in the year between 2004 and 2012 dropped by 36% worldwide, TB is still the leading cause of morbidity and mortality [6]. From world’s 30 highest TB burden countries, Ethiopia ranked seventh and comprises the highest proportion of TB/HIV among SSA countries [7]. Globally, various studies were conducted to assess the incidence and predicators of tuberculosis among HIV-infected patients. For instance, studies conducted in South Africa and Nigeria reveals that the incidence of Tuberculosis was 2.4 and 7.43 per 100 PY respectively [8,9]. Studies conducted in different part of Ethiopia revealed that the incidence of tuberculosis among HIV-infected patients ranging from 3.3 per to 8.6 per 100 PY of observation [2,10]. Studies conducted in Ethiopia and south Africa indicated that being male [11,12], low level of education [13], low baseline CD4 count [14], bedridden functional status [12], WHO clinical stage III and IV [12,14], smoking and alcohol intake [12], were significant predictors of TB among HIV infected patients. On the other hand, INH therapy and cotrimoxazole prophylaxis has shown a protective effect against TB among PLHIV [12].

Ethiopia started the UTT program in 2016 in order to change the distribution of HIV infection [15]. After initiation of UTT program, different intervention strategies have been implemented among PLHIV to reduce the occurrence of TB/HIV co-infection and overcome TB related morbidity and mortality among PLHIV. However, many of deaths occurs due to TB/HIV co-infection and reports regarding the incidence and predictors of tuberculosis among PLHIV after UTT program is limited in Ethiopia. Therefore, the intent of this study is to assess the incidence and predictors of tuberculosis among patients enrolled in Anti-Retroviral Therapy (ART) after UTT program at two public hospitals in Addis Ababa, Ethiopia. The finding of this study will help the health care practitioners, program managers and policy makers to advance TB control and prevention among HIV-infected people.

## Methods

### Study design and setting

A retrospective cohort study design was employed among HIV-positive individuals enrolled at two selected hospitals in Addis Ababa between January 1, 2016, and August 25, 2020. Addis Ababa is the capital of Ethiopia with a total population of 4,794000. There are 12 governmental hospitals in Addis Ababa. Among these, St. Peter specialized hospital and Zewditu memorial hospital are the leading, excellence and provide more ART service. From January 1,2016 to August 25,2020 there were 1479 and 1589 PLHIV on ART at St. Peter specialized hospital and Zewditu memorial hospital respectively. The hospitals also provide palliative care, HIV counseling and testing, STI services and post-exposure prophylaxis (PEP) services.

### Population and sample size determination

All HIV-positive adults (≥15 years) on ART at Addis Ababa and who had at least 1 month of ART follow-up since the UTT program were the target population. Besides, patients who started ART in the selected facilities between January 1, 2016, to August 25, 2020 were the study population. HIV patients who have already developed TB before ART initiation, transferred in from other institution, with unknown date of initiation of treatment and outcome occurrence date were excluded from the study.

The sample size was determined using power cox command of Stata 14 software. Factors such as CD4 count, functional status, sex, and WHO clinical staging were used to estimate the sample size and final sample size was found to be 539. To select the study participants, medical records of all HIV-positive adults on ART from January 1, 2016, to August 25, 2020, were sorted. Then, records were selected using a simple random sampling technique through computer-generated numbers.

### Variables

The dependent variable of this study was the incidence of TB among HIV positive patients. Incident TB case, which is an event of interest, defined as the occurrence of TB cases after ART initiation which is bacteriologically confirmed (with at least one positive AFB microscopy, Xpert MTB/Rif assay positive, or culture positive) or based on clinical decision of expert clinician by analyzing the supportive evidences (suggestive of TB) during follow up. The first group of predictors assessed was socio-demographic characteristics including age, sex, marital status, residence, level of education, and occupation. The second was baseline clinical and laboratory characteristics including WHO clinical stage, CD4 cell count, hemoglobin level, history of TB, and history of opportunistic infections (OIs) and body mass index (BMI). Lastly ART and other medication-related characteristics such as (type ART regimens, regimen change, ART side effects, ART adherence, IPT, and CPT were collected). Occurrence of TB after initiation of ART was considered as event, while, a patient was classified as censored if he/she lost to follow-up, died or didn’t develop TB at the end of the study. These elements were ascertained by reviewing patient records. In this study, lost to follow-up was defined as an HIV-positive patient missing an ART appointment for one to 3 months. Additionally, adherence was classified as good, fair, or poor, according to the percentage of drug dosage calculated from a monthly total dose of ART drugs; hence, good was reported if equal to or greater than 95% or ≤ 3 doses missing per month, fair if 85–94% or 4–8 dose missing per month, or poor if less than 85% or ≥ 9 doses missing per month [15]. Furthermore, low hemoglobin level was defined as hemoglobin level less than to10 g/dl. Finally, opportunistic infections were diagnosed if HIV-positive adults developed any morbidities after starting ART, as documented by the health care professionals.

### Data collection tool and procedures

Data were collected retrospectively from the patient charts and registration book of HIV-positive individuals newly registered at ART clinics during the period January 1, 2016, to August 25, 2020. Data was extracted over 1 month from November 1 to 30, 2020. The data extraction check list was prepared from the ART entry and follow-up forms and the study objectives. Three BSc nurses who have been working in the ART clinics of the selected hospitals collected the data. To ensure data quality, two days of training was given to the data collectors and supervisors about the ways of data extraction. The checklist was pretested before the data collection and amendments were made accordingly. Furthermore, the supervisor and principal investigators performed a strict follow-up and supervision throughout the entire data collection period.

### Data processing and analysis

Data was entered using EPI-DATA Version 3.1 and analyzed using STATA version 14 statistical software. The patient’s follow-up characteristics were described for median, interquartile range and frequency distribution table. Separate graph of Kaplan Meier survival functions and log-rank test was estimated for each categorical variable to compare the survival between different exposure groups. The proportional hazard assumption was checked using Schoenfeld residual test and log [- log] (survival probability) versus log of survival time plot. Bivariable\ Cox-proportional hazard regression model was used to screen variables for the final model. Variables having a p-value of ≤0.20 in the bi-variable analysis were fitted into the multivariable Cox-proportional hazard regression model. Finally, an adjusted hazard ratio with its corresponding 95% confidence interval was reported to declare the presence of a significant association between the explanatory and outcome variables. The model goodness of fit was assessed using Cox-Snell residual plot. This plot calculates an empirical estimate of the cumulative hazard function-based on the Kaplan–Meier survival estimates taking the Cox–Snell residuals as the time variable. If the model fits the data, the plot should be a straight line with a slope of 1.

### Ethical issues

Ethical approval was obtained from ethical review committee of University of Gondar College of Medicine and Health Sciences. Besides, a permission letter was also obtained from the respective hospitals. As the study was conducted through a review of records, the ethics committee waved the need for informed consent. The patient records were accessed from November 1 to 30, 2020 and all the data were fully anonymized before data collection and confidentiality was maintained in all phases of research activities.

## Results

### Socio-demographic characteristics

Among 539 HIV-positive adults’ records reviewed, 529 (98%) records were included in the final analysis while 10 records were excluded because they didn’t contain the required information. Of the total study participants more than half were females 303 (57%). The median age of the participants was 37 (IQR = 29–45) years. Of the total sample 243(46%) were married and the majority 370 (70%) of study participants were orthodox. Concerning educational status almost half of participants have a secondary level of education 267(50.47%). Besides, majority of the participants were from urban areas 519(98%) and 237(45%) were engaged in private works (Table 1).

**Table 1:** Base line socio-demographic characteristics for adult HIV patients on ART in St. peter specialized hospital and Zewditu memorial hospital between January 1,2016 and August 25,2020.

Variables	Category	Frequency	Percentage
Sex	Male Female	226 303	42.7 57.3
Age	15–24 25–34 35–44 ≥45	60 142 182 145	11.3 26.8 34.4 27.5
Marital status	Single Married Divorced Widowed Separate	115 243 131 24 16	21.7 45.9 24.8 4.5 3.0
Residence	Urban Rural	519 10	98.1 1.9
Educational status	No education Primary Secondary Tertiary and above	33 173 267 56	6.2 32.3 50.5 11
Occupation	Unemployment Daily labor Governmental -employee Private House wife	81 13 144 237 14	16.5 2.7 29.4 48.5 2.9
Religion	Orthodox Muslim Protestant others*	370 91 58 10	69.9 17.2 11.0 1.9

others*: Catholic and Judaism.

### Clinical and treatment related characteristics

Of the total participants, 170 (32.14%) had a CD4 count of less than 200cells/ml at baseline and the median CD4 count was 289 and IQR (200-438cells/ml). More than half 298(56.33%) of the participants were WHO stage I at the baseline. Almost half of participants 266(50.28%) were started Cotrimoxazole prophylaxis therapy (CPT). Of the participants 205 (38.75%) had a history of at least one type of opportunistic infections during their presentation. Around two-thirds of the participants were working functional status and had normal weight 333 (62.95%) and 337(63.71%) respectively. Regarding their initial ART regimen, more than three quarter 402 (75.99%) of study participants took the TDF-3TC-EFV-based regimen (Table 2).

**Table 2:** Clinical and treatment related characteristics of adult HIV patients on ART in St. peter specialized hospital and Zewditu memorial Hospital between January 1, 2016 and August 25, 2020.

Variables	Categories	Frequency	Percentage (%)
Baseline CD4 count (In cells/ul)	≤200 >200	170 359	32.1 67.9
Baseline WHO stage	Stage I Stage II Stage III/ IV	298 114 117	56.3 21.6 22.1
Last known WHO stage	Stage I Stage II Stage III/ IV	417 76 36	78.8 14.4 6.8
CPT prophylaxis	Yes No	266 263	50.3 49.7
IPT prophylaxis	Yes No	208 321	39.3 60.7
Types of OI at enrollment (n = 205)	Zoster Toxoplasmosis Bacterial pneumonia Cryptococcus meningitis PCP Others*	38 25 100 14 26 2	18.5 12.2 48.8 6.8 12.7 1.0
TB status	Positive Negative	74 455	14.0 86.0
Functional status	Working Ambulatory Bedridden	333 147 49	62.9 27.8 9.3
BMI	<18.5 18.5–24.9 ≥25	174 337 18	32.9 63.7 3.4
Adherence	Good Fair Poor	388 74 67	73.3 14.0 12.7
Hgb/Baseline/	<10g/dl >10g/dl	68 461	12.9 87.1
Comorbidities at enrollment	Yes No	100 429	18.9 81.1
Types of regimens at start	1c (AZT-3TC-NVP) 1d (AZT-3TC-EFV) 1e (TDF-3TC-EFV) 1j (TDF-3TC-DTG)	80 26 402 20	15.2 4.9 76.1 3.8
Regime change during follow up	To first line To second line Unchanged	355 50 124	67.1 9.5 23.4
Reason for switch first regimen (n = 405)	Toxicity/side effect New drug available Pregnancy immunological failure virological failure	16 351 1 10 27	4.0 86.6 0.2 2.5 6.7
Type of TB	PTB EPTB	66 8	89.0 11.0
TB case definition	New Re-treatment	69 5	93.0 7.0
Viral load	<150(undetectable) >150	345 184	66.0 36.0

Others*: Diarrhea and oral trash.

### Incidence of tuberculosis

**Fig 1:**
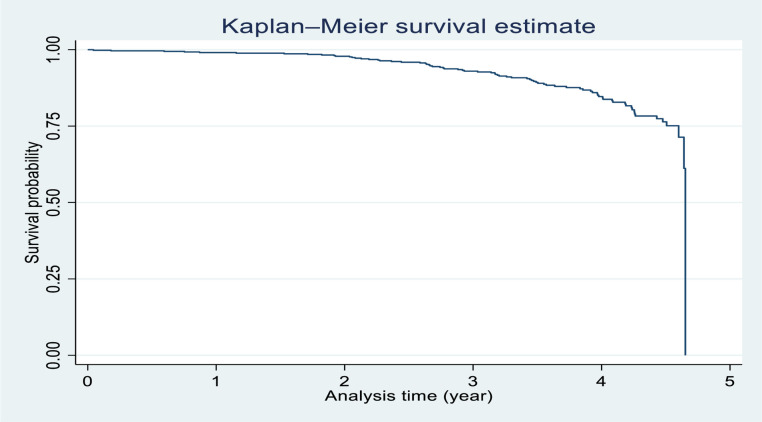
Cumulative survival probability to develop TB with time among patients on ART in St. peter specialized hospital and Zewditu memorial Hospital between January 1, 2016 and August 25, 2020.

Five hundred twenty-nine study participants were followed for different periods in four years and six months which produced 1529 PY of observation. Within the follow-up period, 74 patients were found to develop TB which gives an overall incidence density of 4.84 cases (95% CI:3.83–6.11) per 100 PY. Study participants stayed in the follow-up for a minimum of 0.53 months and a maximum of 56.6 months. The cumulative probability of survival of a patient at the end of 1, 2, 3, and 4 years was 0.97,0.93, 0.89, and 0.85, respectively (Fig 1).

### Predictors of tuberculosis

#### Non-parametric comparison of survival

**Fig 2:**
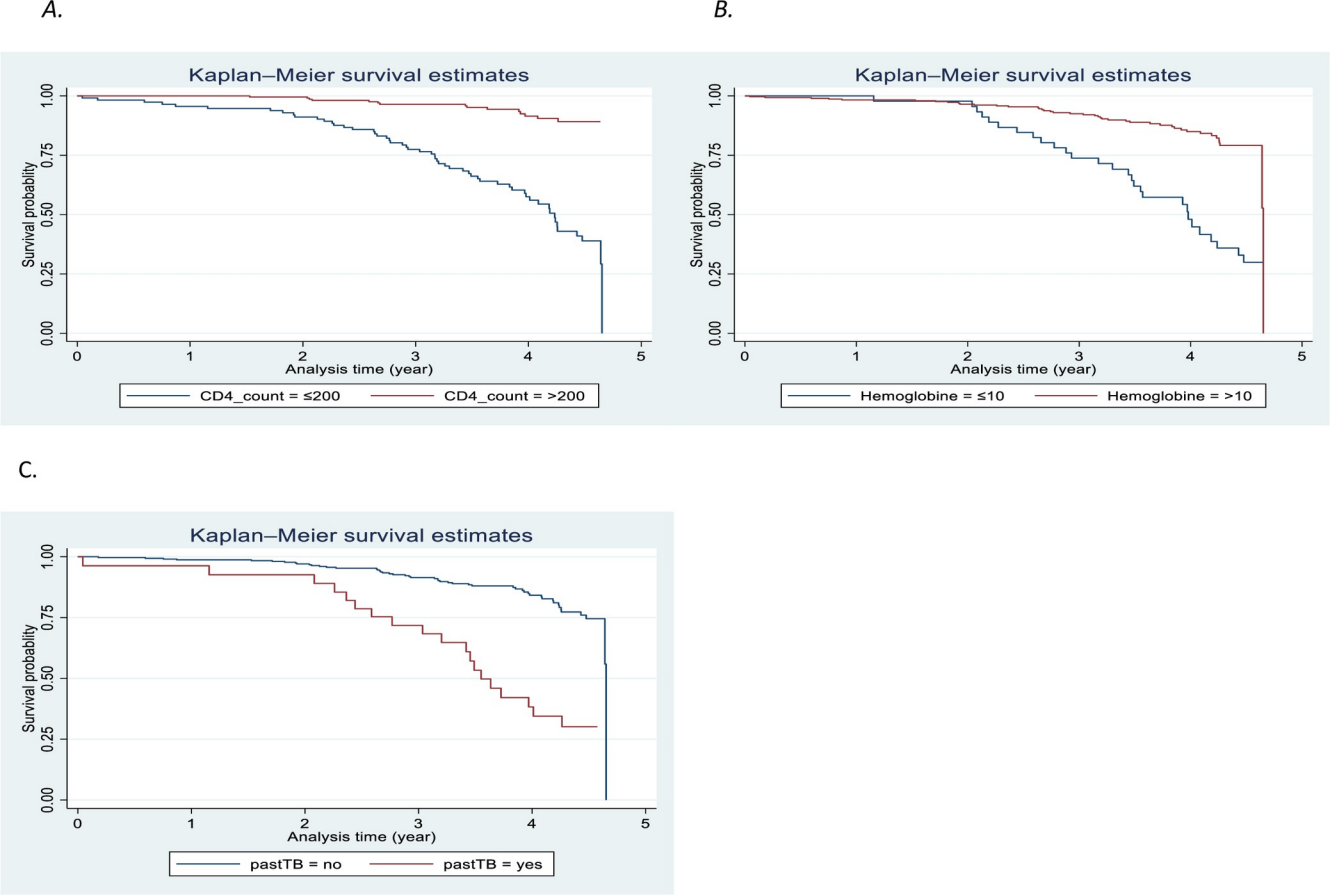
Non-parametric estimates of cumulative survival curves across categories of CD4 count (A), Hemoglobin (B) and Past history of TB (C).

Based on the Kaplan-Meir survival curve, patients with high CD4, no past history of TB, and high hemoglobin were found to have better survival than their counter parts (Fig 2).

#### Cox-proportional hazard model

In multivariable Cox regression analysis, CD4 count, adherence level, BMI, IPT prophylaxis, functional status, and baseline WHO stage were found to be significantly associated with the outcome. The hazard of tuberculosis was 3.14 times higher among participants with CD4 count <200 compared to their counterparts. (AHR = 3.14; 95%CI: 1.64–7.10). The hazard of TB was 2.16 times higher among participants who had poor adherence to treatment compared to their counterparts (AHR = 2.16; 95%CI:1.21–3.85). The hazard of tuberculosis was 2.42 times higher among participants who are underweight as compared to those with a normal weight (AHR = 2.42; 95%CI: 1.30–4.52). Participants who didn’t take IPT prophylaxis had 2.78 times increased hazard of having TB as compared to those who are taking IPT prophylaxis (AHR = 2.78, 95% CI: 1.06–7.30). The hazard of tuberculosis among bedridden patients was three times higher than working (AHR = 3.06; 95%CI:1.50–6.24). Participants who were in WHO stage three or four at baseline had 2.33 times increased hazard of TB compared to those in stage one (AHR = 2.33; 95%CI:1.08,5.02) (Table 3).

**Table 3:** Predictors of Tuberculosis among Adult HIV patients on ART at St. peter specialized hospital and Zewditu memorial Hospital between January 1, 2016 and August 25, 2020.

Independent variables		Survival status		Tuberculosis	
		Event	Censored		
**Variable**	**Category**			**CHR (95% CI)**	**AHR (95%CI)**
Hemoglobin level	>10 g/dl <10 g/dl	42 28	419 40	1.00 4.32(2.67–6.99)	1.00 1.55(0.91–2.63)
Baseline CD4 count (In **cells/ul**)	≤200 >200	57 17	113 342	1.00 7.66(4.31–13.60)	1.00 3.14(1.64–7.10) **
Adherence (last known)	Good Fair Poor	39 5 30	349 69 37	1.00 0.66(0.26–1.70) 5.14(3.14–8.41)	1.00 0.45(0.16–1.24) 2.16(1.21–3.85)*
TB contact	No Yes	64 10	423 32	1.00 2.03(1.00–4.10)	1.00 1.60(0.74–3.44)
TB treatment history	No Yes	52 22	436 22	1.00 4.56(2.68–7.76)	1.00 0.75(0.40–1.43)
Body mass index (BMI) in kg/m^2^	18.5–24.9 >25 <18.5	47 25 2	127 312 16	1.00 1.63(0.38–700) 4.63(2.75–7.75)	1.00 1.97(0.43–8.94) 2.42(1.30–4.52)*
IPT prophylaxis	Yes No	6 68	204 251	1.00 7.41(2.98–18.43)	1.00 2.78(1.06–7.30)*
Functional status (Baseline)	Working Ambulatory Bedridden	24 17 33	309 130 16	1.00 1.64(0.86–3.13) 12(6.94–21.09)	1.00 0.67(0.30–1.49) 3.06(1.50–6.24)*
WHO Stage(baseline)	Stage I Stage II Stage III/ IV	20 5 49	278 109 68	1.00 0.77(0.22–1.95) 7.56(4.33–13.23)	1.00 0.77(0.23–2.52) 2.33(1.08–5.02) *
Co morbidity status	No Yes	43 31	386 69	1.00 3.40(2.10–5.51)	1.00 1.09(0.63–1.89)
OI(at enrollment)	No Yes	26 48	286 169	1.00 2.93(1.77–4.86)	1.00 1.55(0.92–2.63)

** = p value≤0.01

* = p value<0.05.

## Discussion

This retrospective cohort study assessed the incidence and predictors of TB among HIV-positive adults at St. Peter specialized and Zewditu memorial hospitals. The incidence of TB was found to be 4.84 per 100 PY of observation. This finding is in line with studies conducted in the Gurage zone, Ethiopia (4.79cases per 100 PY), Arba Minch, Ethiopia (5.36 cases per 100 PY), and Tanzania (4.4 cases per 100 PY) [16–18]. However, it is higher than the studies from Brazil and the USA which reported the incidence of TB to be (2.28 cases per100 PY), and (0.7cases per 100PY), respectively [19,20]. This might be due to the differences in sociodemographic characteristics of study participants, low healthcare coverage, and high burden of HIV in the study setting. Besides, our study was conducted at specialized hospitals providing tertiary level services for patients referred from health centers or general hospitals. Hence, patients in these hospitals might have advanced disease stage which would increase the risk of developing TB [21]. On the other hand, the incidence was lower than the studies conducted in Afar, Ethiopia (8.6 cases per 100 PY) and Gondar, Ethiopia (7.896 cases per 100 PY) [2,5]. This variation could be explained by the difference in the study period. This means our study was done after the universal test and treat program and this might decrease the incidence [16] as a result of early initiation, strong follow up, and proper implementation in TB/HIV care service. Moreover, creation of better awareness regarding the prevention of TB/HIV co-infection might also contribute to the low incidence [22,23].

From multivariable Cox regression analysis, variables low CD4 count, poor adherence level, low BMI, not taking IPT prophylaxis, bedridden functional status, and being on baseline WHO stage III or IV were found to be significantly associated with the outcome variable.

In the present study, a patient with a base line CD4 count of less than 200 cells/ul is 3.41 times more likely to have TB than their counter parts. This is supported by studies done elsewhere in the world [5,17,24]. This could be attributed to HIV often causes immunosuppression directly by reduction of CD4 count, because of progressive impairment of cellular immunity. This leads to decrement of the body’s defense ability and enable the disease (HIV) progresses to an advanced stage. Lastly, the patient might develop more serious diseases including TB [25,26].

According to this study, participants with poor adherence to the treatment had an increased hazard of having TB compared to their counterparts This is in agreement with another study done in Ethiopia [27]. This can be explained by patients with poor adherence to ART are prone to rapid viral replication which worsen immunological and clinical outcomes. This leads to declining of CD4 cell count which will end up with high risk of developing opportunistic infections including TB [28,29].

This study identified that being underweight was found to increase the hazard of TB compared to their counterparts. This finding is consistent with that of different studies done in Ethiopia, Tanzania, and South Africa [5,30,31]. This might be due to the fact that HIV patients frequently encountered symptoms like loss of appetite and decreased intake of food, this leads to undernutrition which further weakens the immunity level. As a result, this patients will be more at risk to develop both primary and secondary TB [5,32].

In the present study, participants who didn’t take IPT prophylaxis had 2.78 times increased hazard of having TB. This result is in line with studies conducted in Brazil and Ethiopia [20,21]. IPT has a significant role in eradicating latent tuberculosis and prevention of new infections which might decrease the incidence of active TB cases [20].

Being bedridden was also found to increase the hazard of tuberculosis among HIV- positive adults compared to their counterpart. This finding corresponds with different studies conducted in Ethiopia [32–34]. This could be attributed to the fact that being bedridden is an indicator of advanced disease. Hence, this patients are prone to malnutrition, inability to perform daily tasks and lack of physical activity, that will eventually lead them to have week immunity and tuberculosis infection [33].

According to this study, advanced WHO clinical disease stage (III and IV) was found to increase the hazard of TB among HIV-positive patients. This result is similar to studies done in Ethiopia, Tanzania, and South Africa [18,33,35]. This could be explained by patients in advanced WHO stage are at increased risk of having primary or secondary TB infection due to their compromised immunity [5].

This research was conducted after the universal test and treat (UTT) program was established in Ethiopia. Thus, the finding of this study will bring new pieces of evidence on the effect of UTT in a clinical setting. However, there are some limitations of this study such as due to the secondary nature of the data some important variables like housing conditions, family size, household incomes, and substance use were not included. In addition, since this study was facility-based it does not capture HIV-positive individuals who are out of care (at the community level).

## Conclusion

In this study, the incidence of tuberculosis is slightly low comparing to the previous studies done before the universal test and treat program. Patients who had low CD4 (<200 cell/ul), with a poor level of adherence, BMI<18.5kg/m2, did not take IPT prophylaxis, bedridden functional status, and baseline WHO stage III or IV are associated with TB co-infection among adult HIV positives. Hence, it is better to improve the package of care services, early screening and diagnosis for patients who have the risks at enrollment. We recommend to do community and facility based active TB case detection on this high-risk groups using health extension workers and health professionals in the facilities. Furthermore, it is better to conduct prospective study to alleviate the problem of incomplete records and incorporate social, economic, and behavioral factors.

## Abbreviations

AFB: Acid Fast Bacilli
AHR: Adjusted Hazard Ratio
AIDS: Acquired Immunodeficiency Syndrome
ART: Anti-Retroviral Therapy
CI: Confidence Interval
CPT: Co-trimoxazole Preventive Therapy
EDHS: Ethiopian Demographic Health Survey
HAART: Highly Active Antiretroviral Therapy
HIV: Human Immunodeficiency Virus
INH: Isonicotinic acid Hydrazide
IPT: Isoniazid Preventive Therapy
MTB: Mycobacterium Tuberculosis
OI: Opportunistic infections
PLHIV: People Living with Human Immunodeficiency Virus
PTB: Pulmonary Tuberculosis
PY: Person Year
TB: Tuberculosis
TB/HIV: HIV-related Tuberculosis
UNAIDS: United Nation Program of HIV/AIDS
UTT: Universal Test and Treat
